# Copper Induced Conformational Changes of Tripeptide Monolayer Based Impedimetric Biosensor

**DOI:** 10.1038/s41598-017-10288-z

**Published:** 2017-08-25

**Authors:** Evgeniy Mervinetsky, Israel Alshanski, Yonatan Hamo, Leonardo Medrano Sandonas, Arezoo Dianat, Jörg Buchwald, Rafael Gutierrez, Gianaurelio Cuniberti, Mattan Hurevich, Shlomo Yitzchaik

**Affiliations:** 1 Institute of Chemistry, The Hebrew University of Jerusalem, Safra Campus, Givat Ram, Jerusalem, 91904 Israel; 2 Center for Nanoscience and Nanotechnology, The Hebrew University of Jerusalem, Jerusalem, 91904 Israel; 3 Institute for Materials Science and Max Bergmann Center of Biomaterials, TU Dresden, 01069 Dresden, Germany; 4 Max Planck Institute for the Physics of Complex Systems, 01187 Dresden, Germany; 5 Dresden Center for Computational Materials Science, TU Dresden, 01062 Dresden, Germany; 6 Center for Advancing Electronics Dresden, TU Dresden, 01062 Dresden, Germany

## Abstract

Copper ions play a major role in biological processes. Abnormal Cu^2+^ ions concentrations are associated with various diseases, hence, can be used as diagnostic target. Monitoring copper ion is currently performed by non-portable, expensive and complicated to use equipment. We present a label free and a highly sensitive electrochemical ion-detecting biosensor based on a Gly-Gly-His tripeptide layer that chelate with Cu^2+^ ions. The proposed sensing mechanism is that the chelation results in conformational changes in the peptide that forms a denser insulating layer that prevents RedOx species transfer to the surface. This chelation event was monitored using various electrochemical methods and surface chemistry analysis and supported by theoretical calculations. We propose a highly sensitive ion-detection biosensor that can detect Cu^2+^ ions in the pM range with high SNR parameter.

## Introduction

Monitoring the concentration of Cu^2+^ ions in aqueous solution is an imperative issue in environmental and biological sciences^1^. The typical concentration of copper ions in human blood serum valuates about 20 μM. Fluctuations by 1–2 μM in copper ion concentrations in biofluids and tissues are associated with different diseases, including Menkes syndrome, myeloid leucosis, liver cirrhosis and Wilson’s disease^2^.

Standard methods like atomic absorption and elementary analysis are commonly applied to detect and monitor metal ions, among them copper ions, concentration. These classical methods are highly accurate, but are also expensive, non-portable, time-consuming and requires handling by highly trained personal^3, 4^. There is a growing interest in the development of portable and highly sensitive electrochemical sensors that will provides an easy and rapid quantification of metal ions^5^. Electrochemical Impedance Spectroscopy (EIS) is high-sensitive, non-labeled electrochemical technic, reported as efficient sensing method for detecting C-reactive protein^6^, organic pollutants^7^ and inorganic ions^7, 8^.

Previous researches in our group have showed that the properties of Self-Assembled Monolayers (SAM) can be modified as a result of changes in their structure, including their electrical properties^9–13^. These studies suggest that the conformational changes in monolayers could affect the density and the ability of ions to penetrate the layer, which affects charge transfer. This dependence was demonstrated using Electrochemical Impedance Spectroscopy^10, 12, 13^, and Ion-Sensitive Field Effect Transistor (ISFET) analysis^11^.

The Gly-Gly-His (GGH) tripeptide is a known chelator of Cu^2+^ ions^1, 5, 14–16^. Several studies demonstrated that gold surface grafted with GGH to can be used for Cu^2+^ sensing^1, 5, 14–19^. These studies used stepwise indirect grafting strategy of GGH to gold substrates. The strategy includes grafting of functional thiols as a linker and only later the GGH attached to the surface through the linker^5, 14–19^.

We present here a direct approach for the preparation of SAM of GGH on gold surface using a synthesized the unified lipoic acid-tripeptide conjugate (Lpa-GGH), enabling grafting of the peptide SAMs directly onto the gold surfaces.

This new Au-GGH SAM was used to study/characterize the structural changes of the peptide upon metal complexation by different characterization methods, including electrochemistry X-ray photoelectron Spectroscopy (XPS) and Atomic Force Spectroscopy (AFM).

Our results demonstrates that alteration in the environment, attained by varying the Cu^2+^ concentration, results in a conformation change of the GGH peptide on the surface. The resulting denser SAM interferes with the charged species transfer to the surface that can be indicated by an increase of the impedimetric signal. This allowed us to prepare an ultra-sensitive GGH based biosensor for copper that is very easy to handle and analyze.

## Experimental

All solutions used in this work were prepared with Milli-Q Water (18.3 MΩ/cm, Millipore Milli-Q system (Bedford, MA). Buffer solutions used were 50 mM ammonium acetate (pH = 7.0). The synthesis of the tripeptide GGH functionalized with lipoic acid was performed using standard Fmoc SPPS chemistry and HATU as a coupling agent^20^. The peptide was then cleaved from the solid support using TFA cleavage solution and purified using RP-HPLC and analyzed using analytical HPLC (Figure S2) and mass spectrometry (Figure S1).

Electrochemical analyses were conducted with Metrohm- Autolab PGSTAT-12 digital potentiostat (EcoChemie BV, Utrecht, The Netherlands) connected to Nova software for utilizing Electrochemical Impedance Spectroscopy (EIS), Cyclic Voltammetry (CV) and Square Wave Voltammetry (SWV). A three-electrode cell was used for the measurements: Ag/AgCl (in 3 M KCl) as reference electrode (RE), Pt as counter electrode (CE) and Au as working electrode (WE). Polycrystalline bulk gold electrodes with a 2 mm diameter were used for electrochemical measurements (CH instruments). These electrodes were manually-polished on micro-cloth pads (Buehler, Lake Bluff, IL) with de-agglomerated alumina suspension (Buehler) of decreasing particle size (1.0 and 0.05 mm). After polishing the electrodes were sonicated in triple distilled water (TDW) for 10 min followed by additional sonication in EtOH for 10 min. Subsequently, Au WE were cleaned electrochemically by CV in H_2_SO_4_ (0.5 M). Then WE casted by 70 µL 250 μM Lpa-GGH in Ammonium Acetate Buffer solution (pH = 7.0) for 2 h, then rinsed copiously with TDW and dried under dry N_2_ stream. Following peptide assembly the modified electrodes were exposure to 10 μM Cu^2+^ solution (in HNO_3_ 640 μM/Ammonium Acetate buffer solution, pH = 7.0) for 2 h, then rinsed copiously with TDW and dried under dry N_2_ stream. For dose response, adsorption of peptide performed by drop casting (250 μM Lpa-GGH in Ammonium Acetate Buffer, for 2 h), exposure to Cu^2+^ solution (1 pM-100 μM) was conducted by deep casting for 15 min, washing in TDW and next exposing of the same electrode to higher Cu^2+^ concentrations. The EIS characterization was done in EIS solution contained 5.0 mM K3[Fe(CN)_6_], 5.0 Mm K4[Fe(CN)_6_] (RedOx species); and 0.1 M of KCl as supporting electrolyte. The spectra were recorded at a frequency range of 0.1 Hz–10 kHz with amplitude of 10 mV, at the formal potential of the RedOx species vs. Ag/AgCl electrode. The R_CT_ values were fitted to the following equivalent circuit Rs[(R_CT_|W)||CPE]. This equivalent circuit presents good fitting for EIS, when ‘Rs’ value is related for solution resistance, ‘R_CT_’ is the charge-transfer resistance for penetration of RedOx species through the layer. ‘W’ is Warburg parameter related to diffusion of RedOx species to the electrode surface from the solution. ‘CPE’ is Constant Phase Element that related to the layer’s capacitance.

For surface characterization gold layer (100 nm) was evaporated on top of highly-doped n-type Si wafer (<100>, R < 0.003 Ω/cm). Lpa-GGH was adsorbed on these substrates.

XPS spectra were recorded using a monochromated Al Kα X-ray source on a Kratos Axis-HS instrument. XPS analyses were applied to characterize the self-assembled peptide monolayers and the complexation of the peptide with Cu^2+^ ions.

AFM (Bruker, Innova) was executed in order to monitor topography changes of the layer as a result of peptide adsorption and chelation with Cu^2+^ ions. AFM analysis (tapping mode) was performed on bare Au layer evaporated on Si substrate. Lpa-GGH adsorbed to Au layer and AFM topography analysis was done for modified layer. Than the sample was exposed to Cu^2+^ solution and AFM was measured thereafter. Same procedures for adsorption and exposure to Cu^2+^ ions solution were performed to prepare XPS samples.

## Results and Discussion

### Formation and characterization of Gly-Gly-His SAM

The synthesis of the lipoic acid functionalized tripeptide Gly-Gly-His (Lpa-GGH) was performed using a standard Fmoc SPPS chemistry.

**Figure 1:**
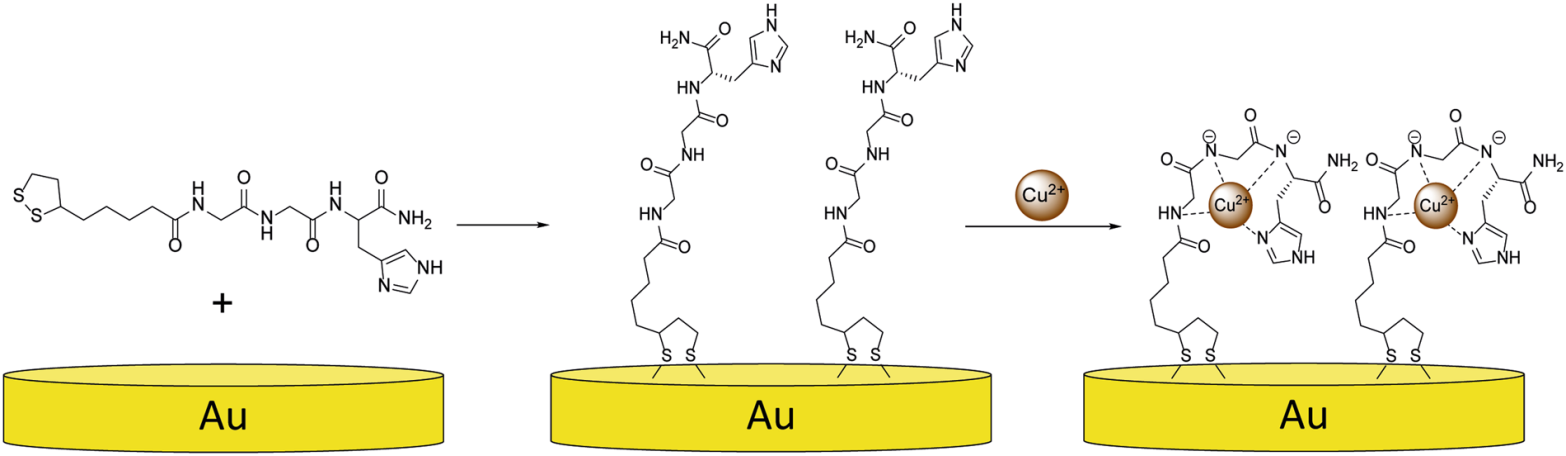
General scheme of peptide monolayer and its complexation with Cu^2+^ ions.

The peptide was adsorbed onto gold surface by formation of self-assembled monolayer as presented in Fig. 1.

The adsorption of the peptide to the gold surface was verified using XPS analysis of the Au-GGH SAM (Figures S3 and S4). The XPS analysis showed a peak with a binding energy (E_BE_) of 161.4 eV (Figure S4). This frequency corresponds to electron emission of sulfur 2p 3/2 that is related to the S-Au bond^21^, a characterizing feature that indicates that Lpa-GGH is absorbed to gold surface. As expected, this peak was absent for bare gold reference surface. The XPS analysis indicated also the presence of C, N, S atoms. This provides further proof for the formation of Au-GGH SAM.

**Figure 2:**
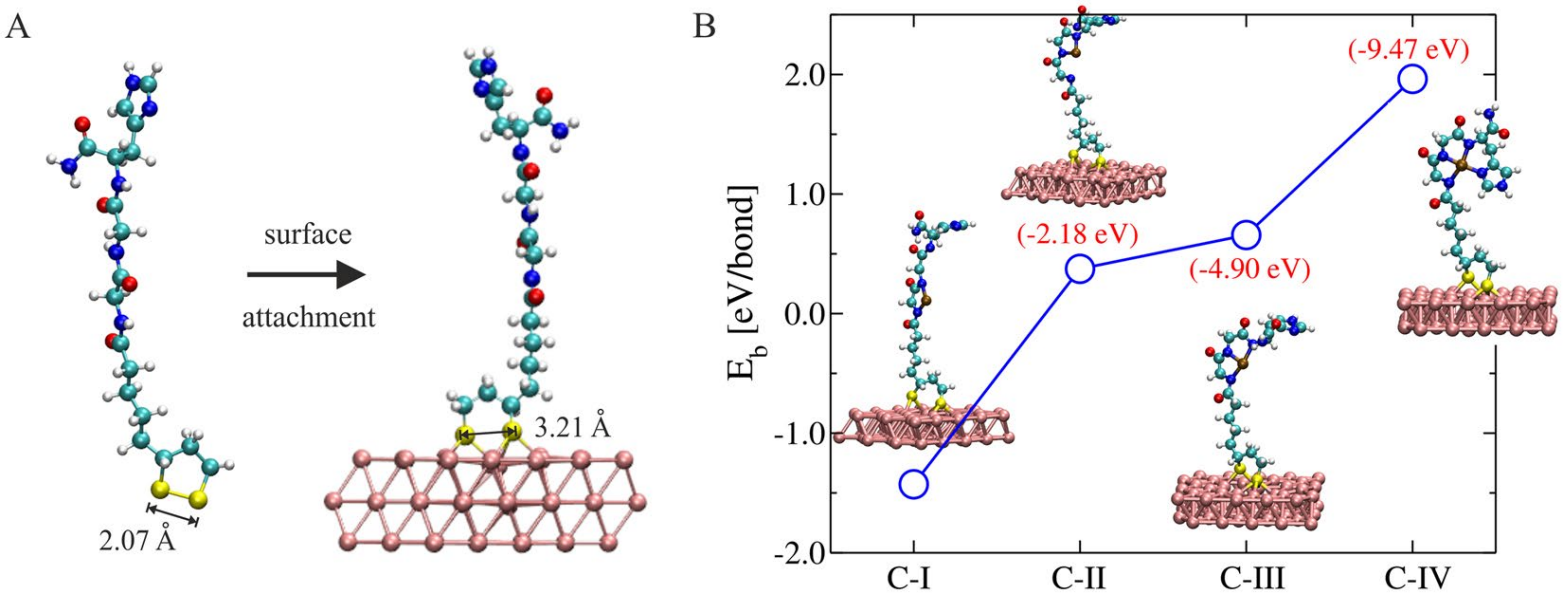
(**A**) Surface attachment of Lpa-GGH onto Au(111). The S-S distance in both cases is highlighted in order to emphasize the bond breaking effect after binding to the surface. (**B**) DFT calculation of the ion binding energy per N-Cu bond for four different conformations of the Lpa-GGH/Au(111) system. The differences of total energy respect to (C-I) are shown in parentheses. The structure with four N-Cu bonds (C-IV) represents the most stable configuration.

To gain a deeper insight into the adsorption process of Lpa-GGH (at pH = 7.0) onto the gold surface, we carried out density functional theory (DFT) calculations for the following structure setup. A gold surface in the crystallographic direction (111) composed by 6 atomic layers and with periodic boundary conditions in all the directions was considered. The dimensions of the supercell in the XY plane are 14.43 Å × 14.95 Å. The DFT calculations of the Au-GGH SAM showed that upon attachment of the molecule and subsequent structural relaxation, the adsorption energy of the molecule to the substrate amounts −0.87 eV. Our calculations further show S-Au bond lengths of 2.45 Å and 2.65 Å indicating that each sulfur atom of the lipoic acid forms covalent bonds with Au atoms. In addition, the calculated S-S distance in the Au-GGH SAM was 3.21 Å, which differs from the S-S bond length of 2.07 Å obtained for the free Lpa-GGH (Fig. 2A). These results indicate that the S-S bond of the Lpa breaks when the molecule interacts with the gold surface and, hence, support the experimental results.

We have further studied the energetic stability of the Au-GGH SAM after the addition of copper ion, Cu^2+^ (the Lpa-GGH molecule was previously threefold deprotonated and structurally optimized). For this, we have taken in account four different ion-binding scenarios as it is shown in Fig. 2B (most favorable structures). All of them display S-S bond breaking with S-S distances between 3.0 Å and 3.25 Å, values which are close to those found for Lpa-GGH/Au(111) without ions. Here, we found that significant structural changes give more stability to the system. Thus, in agreement with experimental findings, we found that when the peptide enfolds the Cu^2+^ and, hence, generates four N-Cu bonds (C-IV), the system is energetically most stable, with a total energy lower by 9.47 eV with respect to C-I (linear shape with two N-Cu bonds) and by 4.57 eV lower than C-III (folded structure with three N-Cu bonds). This four-bonded structural conformation was also experimentally observed by Wawrzyniak *et al*.^18^. Concerning the ion binding energy E_b_, we find that C-IV has the highest binding energy per N-Cu bond of about 1.92 eV. Here, we have defined the ion binding energy as: E_b_ = −(E_mol+Cu/sur_ − E_mol/sur_ − E_Cu_), where E_mol+Cu/sur_, E_mol/sur_, E_Cu_ are the total energies of the whole system, of the Lpa-GGH/Au(111) system, and of an isolated Cu atom, respectively.

The DFT models highlight that the Au-GGH SAM gains stability upon Cu^2+^ binding by adopting a more concise turn conformation with a tetravalent complex core. This is very different from the structure of the peptide prior to the introduction of ions. This suggests that in the presence of Cu^2+^, a denser monolayer might be formed as the peptide tends to block more of the surface compared to the open form of the peptide in the Au-GGH SAM.

### Theoretical modeling

The adsorption of Lpa-GGH onto Au(111) surface and the corresponding interaction properties with Cu^2+^ ions were theoretically addressed at the DFT level. We used mixed Gaussian plane wave (GPW) methods with the standard implementation in the CP2K package^22^. Here, the Kohn-Sham orbitals are expanded into linear combinations of contracted Gaussian type orbitals and complemented by a plane-wave basis set in order to compute the electronic charge density. In all calculations, the PBE exchange-correlation functional was used^23^, and its corresponding norm-conserving pseudo-potential GTH (Goedecker, Teter and Hutter)^24^. Finally, a DZVP (double zeta for valence electrons plus polarization functions) basis set complemented with a plane-wave basis set energy cut-off 350 Ry was employed and dispersion corrections were included through the standard D2-Grimme parameterization^25^.

### Electrochemical characterization

**Figure 3:**
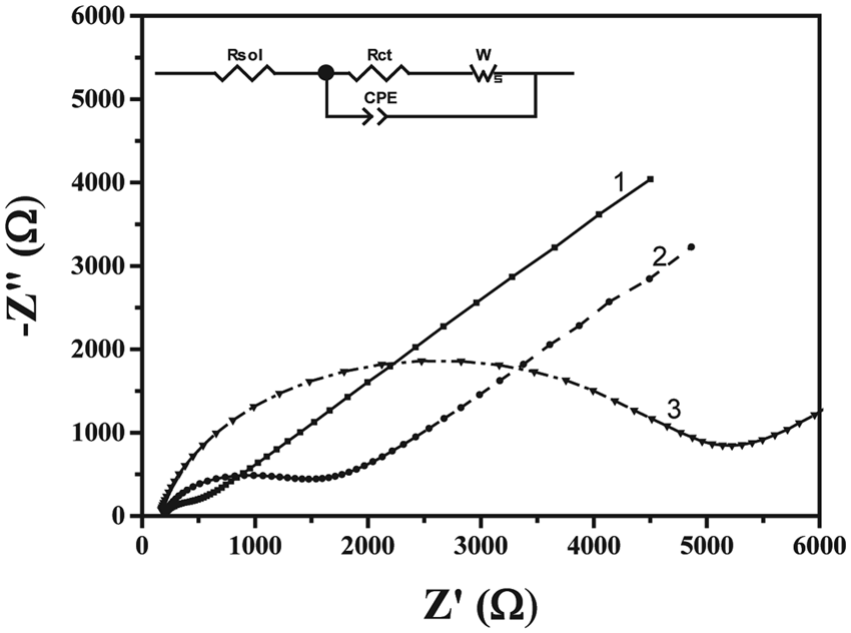
Electrochemical Impedance Spectroscopy. Bare Au electrode (1), Lpa-GGH modified electrode (2) and after chelation with Cu^2+^ ions (3).

EIS measurements were performed at three stages, bare gold electrode, after adsorption of the peptide to the gold electrode and finally after exposure to metal ions. The impedimetric data shows an increase in R_CT_ following the formation of Lpa-GGH monolayer on Au compared to the bare electrodes (Fig. 3). The increase in impedance after the adsorption of the peptide to R_CT_ = 1374 Ω relatively to R_CT_ = 586 Ω of bare Au electrode is associated with the formation of an organic layer that insulate the surface of the electrode. Additional increase for R_CT_ = 5184 Ω was observed after the addition of 10 μM solution of Cu^2+^ (Fig. 3). We suspect that the increase in impedance following the exposure to Cu^2+^ is due to changes in the Au-GGH SAM packing density. Following the theoretical study, we claim that the conformational change of the ligating peptide, from linear free peptide to looped peptide that complexes copper ions transformation leads to a denser peptide insulating layer that prevents the RedOx species from reaching the surface of the electrode. This insulation leads to a decrease in charge transfer, which is reflects in higher R_CT_ values.

**Figure 4:**
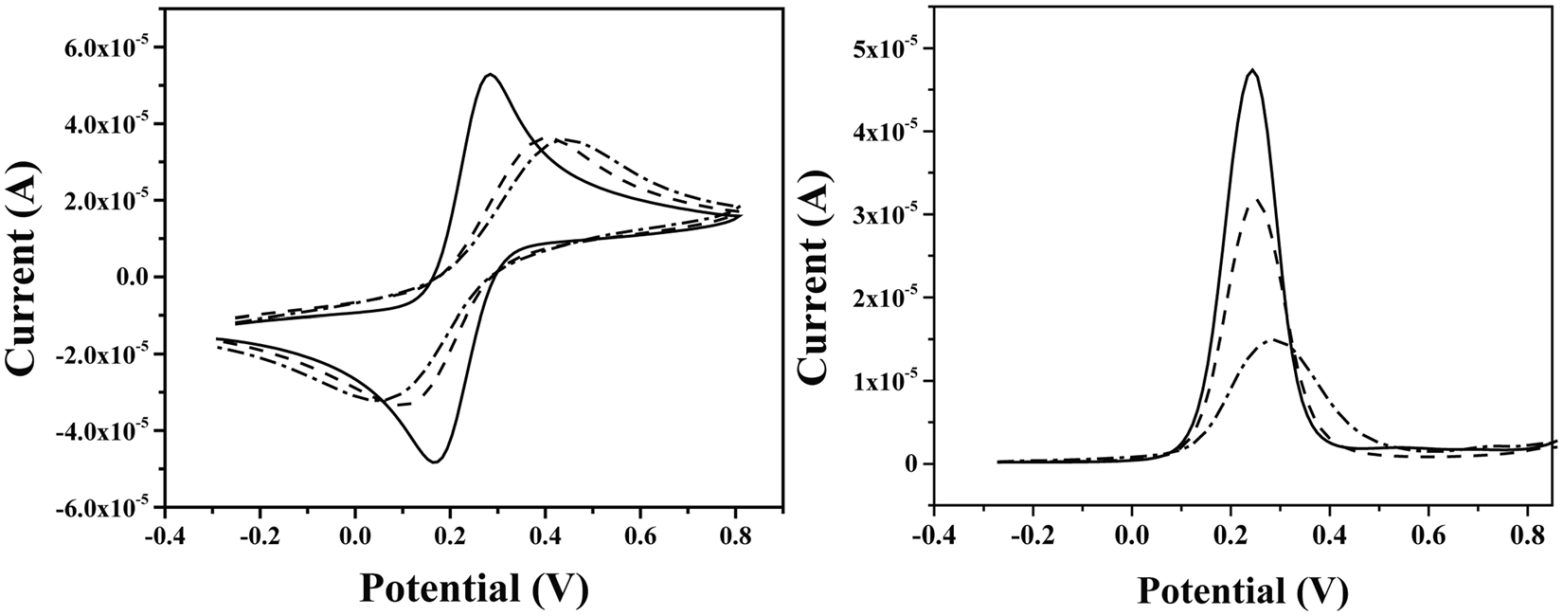
(**A**) Cyclic Voltammetry and (**B**) Square Wave Voltammetry measurements of bare Au electrodes (solid), Lpa-GGH grafted electrode (dash), and Lpa-GGH layer exposure to 10 μM Cu^2+^ solution (dot).

CV and SWV analysis of the same three stages were performed (Fig. 4). Both in CV and SWV show that upon exposure of the peptide-modified electrode to Cu^2+^, the current decreased. Moreover, the SWV data shows a shift of the oxidation peak to more positive potential, as reported previously^26, 27^. This results are in agreement with both the EIS and the theoretical study and can be explained by the conformational changes of the peptide that result in a denser layer that in turn decreases the permeability of the RedOx species to the surface of the electrode. Resulting from the above cascade, higher potentials are necessary for oxidation of the electrode.

### Surface characterization

**Figure 5:**
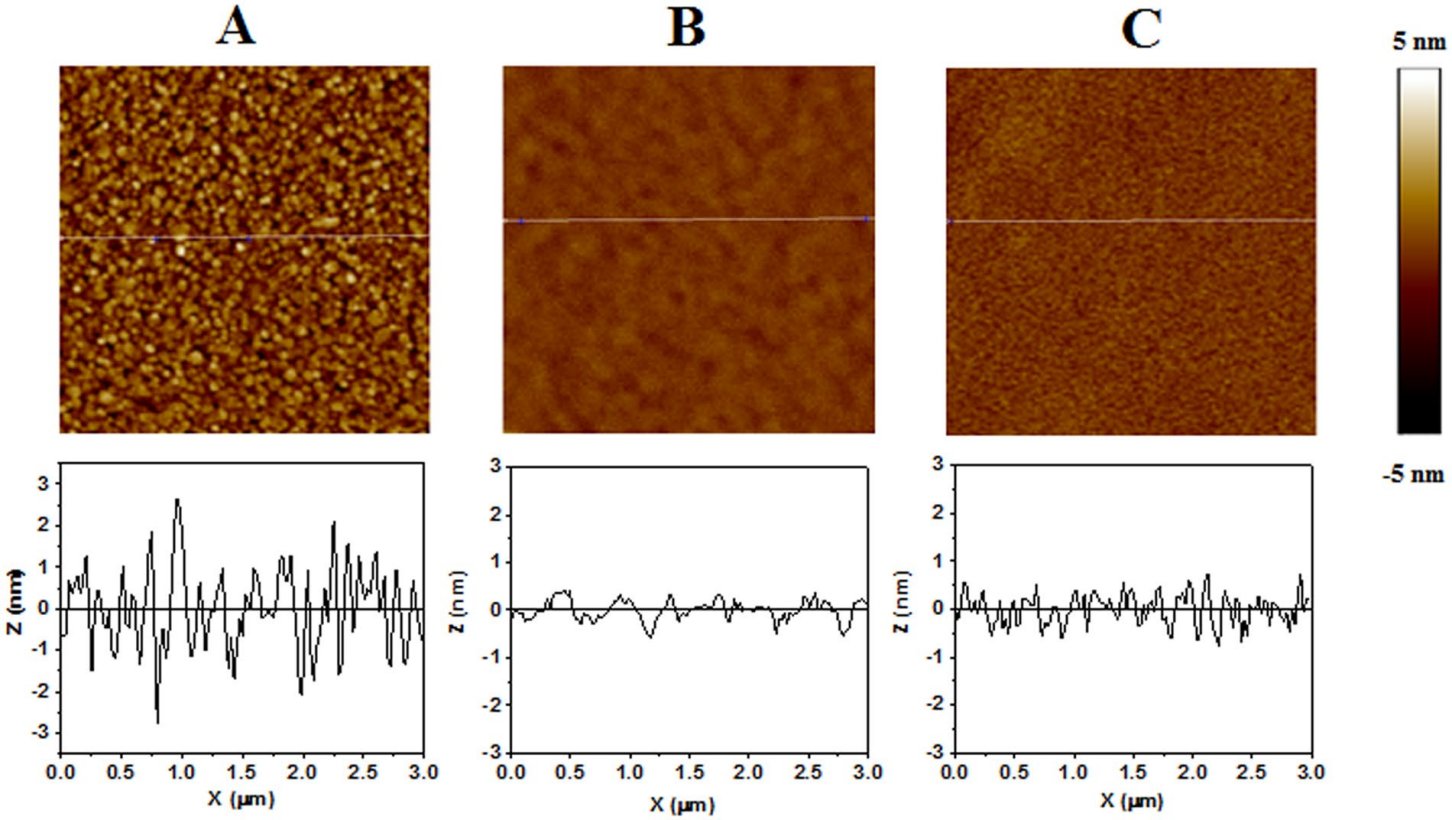
AFM topography (top) and cross-section (bottom) of bare Si-Au surface (**A**); after the adsorption of Lpa-GGH peptide (**B**); and following exposure to Cu^2+^ ions (**C**). Each sample is 3 μm × 3 μm area.

The change in peptide conformation upon exposure to Cu^2+^ is associated with a change in Au-GGH SAM properties; hence, we characterized the surface morphology of the biosensor using AFM. We evaluated the gold surface in each of the following stages: the bare gold surface, the Au-GGH surface before and after exposure to Cu^2+^ ions. The AFM analysis show that after the adsorption of the Lpa-GGH peptide layer, the averaged value of root mean square (RMS) of roughness (ρ) was considered to eliminate local changes in topography.The roughness of the surface decreased from 0.893 nm to 0.177 nm (Fig. 5). This correlates with flatting of surfaces resulting from intermolecular packing of the peptide SAM^17^ and surface energy minimization^9, 10, 12^. After exposure of the peptide layer to Cu^2+^ ions the roughness of the surface increased to 0.284 nm (Fig. 5). This increase in surface roughness is attributed to the conformational changes in the peptide layer after chelation. The AFM analysis hence, also support our hypothesis.

We used XPS to characterize the changes in elemental composition of the peptide layer upon the chelation with Cu^2+^ ions. For the Si-Au-GGH sample binding energy peak observed at 161.4 eV is related to S-Au bond^21, 28^. In addition, the peak at 932.7 eV (Figure S3) corresponds with Cu^2+^ 2p 3/2 binding energy was present only in the spectra of the sample that was exposed to Cu^2+^ ions. The shift in energy from the typical XPS Cu^2+^ peak (933.6 eV) is attributed to the chelation with the GGH peptides, a phenomenon previously reported in literature^29, 30^.

XPS analysis strongly indicate that the adsorption of the Lpa-GGH peptide is accompanied by the disulfide bond breaking and Au-S bond formation as was suggested by the model (Figure S4). Furthermore, the presence of peaks that correlates with copper ions on the Au-GGH SAM proves that the metal is in complex with of the peptide.

### Copper ions dose response using EIS

The results described until now proved that the GGH-Au-SAM is sensitive to Cu^2+^ environment. In order to develop a reliable Cu^2+^ sensor, it was crucial to evaluate the correlation between the concentration of the copper ions and the electrochemical signal of our ligand based SAM.

**Figure 6:**
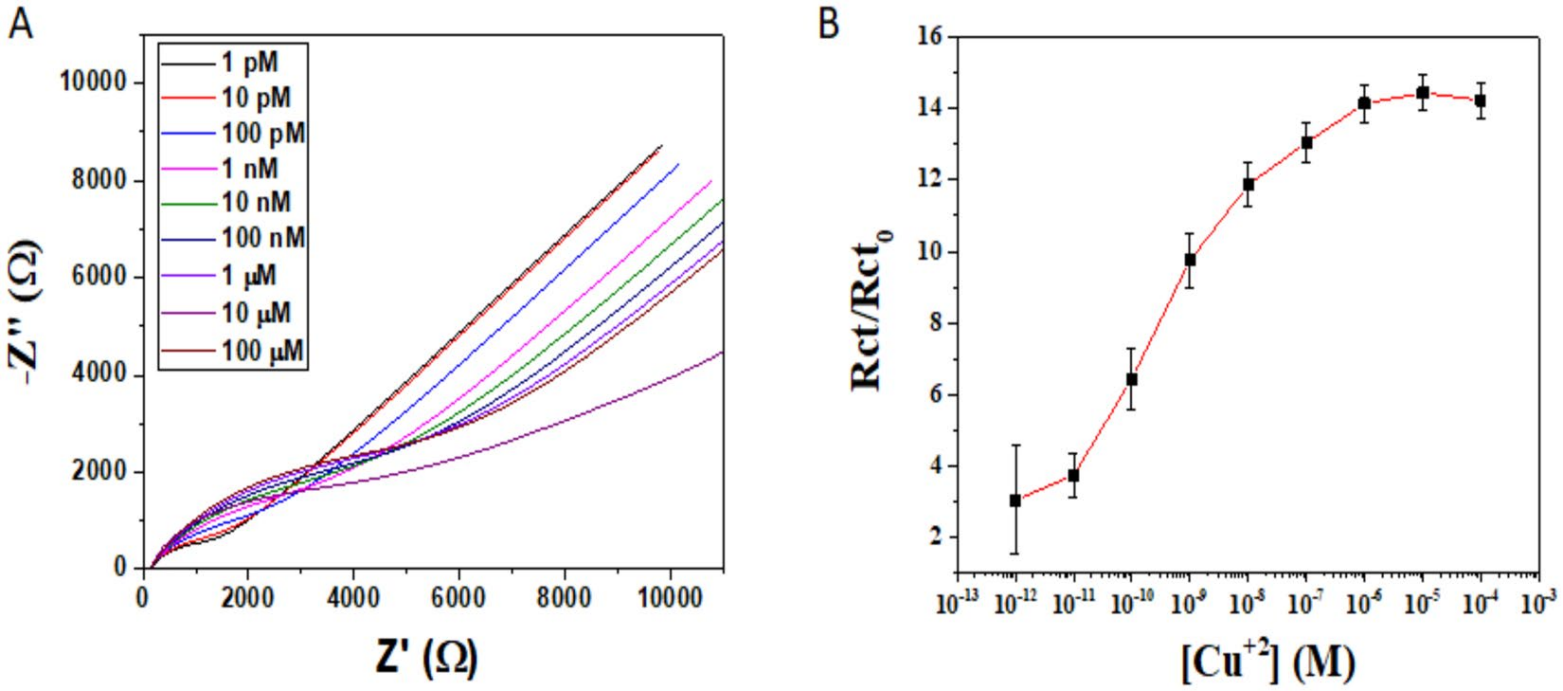
EIS-derived dose response of Cu^2+^ ion concentration: (**A**) Electrochemical Impedance Spectra with different concentrations of Cu^2+^; (**B**) Rct values after exposure to Cu^2+^ solutions normalized by the initial Rct.

To quantify the effect of Cu^2+^ concentration on Au-GGH working electrodes, those electrodes were exposed to increasing concentrations of Cu^2+^ and the response on the SAM in each concentration was analyzed using EIS (Fig. 6).

We normalized the R_CT_ values extracted from the EIS data (R_CT_ by dividing them by the initial R_CT0_ value of the peptide-modified electrode before exposure to Cu^2+^ ions (“initial”) (R_CT_/R_CT0_) (Fig. 6). Based on these experiments, we can determine that the sensitivity range of our biosensor is pM to μM regime and that saturation occurs at concentrations higher than 1 µM. Moreover, the R_CT_ values in the pM range were at least 3 times higher than of the “initial” electrode, while in the μM range it was 14 times higher. Saturation of the system is achieved when no more Cu^2+^ ions can be chelated, possibly due to the fact that are no more peptides available for chelation.

To the best of our knowledge the detection for Cu^2+^ reported in the literature by electrochemical^31^, fluorimetric method and electrothermal atomic adsorption spectrometry is 3, 125 and 100 pM, respectively^19, 32, 33^. The Au-GGH sensor presented here show sensitivity to Cu^2+^ in the sub-nanomolar range and is comparable with the other previously described methods.

## Conclusions

We present a new method for single-step fabrication of GGH self-assembled monolayers on gold surfaces for highly sensitive electrochemical ion sensing. This novel sensing system demonstrates high sensitivity to a wide range of Cu^2+^ ions concentrations: from 1 pM to 10 μM. Self-contained XPS analysis supports peptide chemisorption and peptide-Cu^2+^ ion chelation. In addition, AFM measurements and theoretical calculations support the hypothesis of significant morphological changes in the peptide layer due conformational changes, resulting from chelation.

## Electronic supplementary material

Supplementary Information
