# Supplementary material for: Copper Induced Conformational Changes of Tripeptide Monolayer Based Impedimetric Biosensor
Source: Sci Rep. 2017 Aug 25;7:9498. doi: 10.1038/s41598-017-10288-z (PMC5572728; doi:10.1038/s41598-017-10288-z)
Supplement: Supplementary file 1 — Supplementary Information [file 41598_2017_10288_MOESM1_ESM.pdf]

Copper Induced Conformational Changes of Tripeptide Monolayer Based Impedimetric Biosensor

Evgeniy Mervinetsky[1,2], Israel Alshanski[1,2], Yonatan Hamo[1,2], Leonardo Medrano Sandonas[3,4], Arezoo Dianat[3], Jörg Buchwald[3], Rafael Gutierrez*[,3], Gianaurelio Cuniberti[3,5,6] Mattan Hurevich[1,2], and Shlomo Yitzchaik*[,1,2]

[1] Institute of Chemistry, the Hebrew University of Jerusalem, Safra Campus, Givat Ram, Jerusalem 91904, Israel.

[2]Center for Nanoscience and Nanotechnology, the Hebrew University of Jerusalem, Jerusalem 91904, Israel

[3] Institute for Materials Science and Max Bergmann Center of Biomaterials, TU Dresden, 01069 Dresden, Germany.

[4] Max Planck Institute for the Physics of Complex Systems, 01187 Dresden, Germany.

[5] Dresden Center for Computational Materials Science, TU Dresden, 01062 Dresden, Germany.

[6]Center for Advancing Electronics Dresden, TU Dresden, 01062 Dresden, Germany

Supporting Information

Materials and Characterization of Lpa-GGH synthesis

	m/z found	m/z calculated
(LGGH+H)+	457.1690	457.1687
(LGGH+Na)+	479.1518	479.1506
(LGGH+K)+	495.1459	495.1246

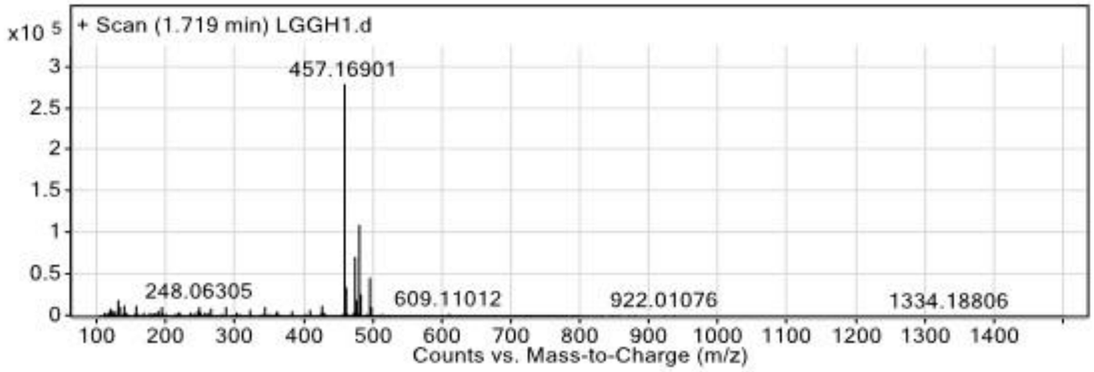

Figure S1: MS Characterization of synthetic Lpa-GGH

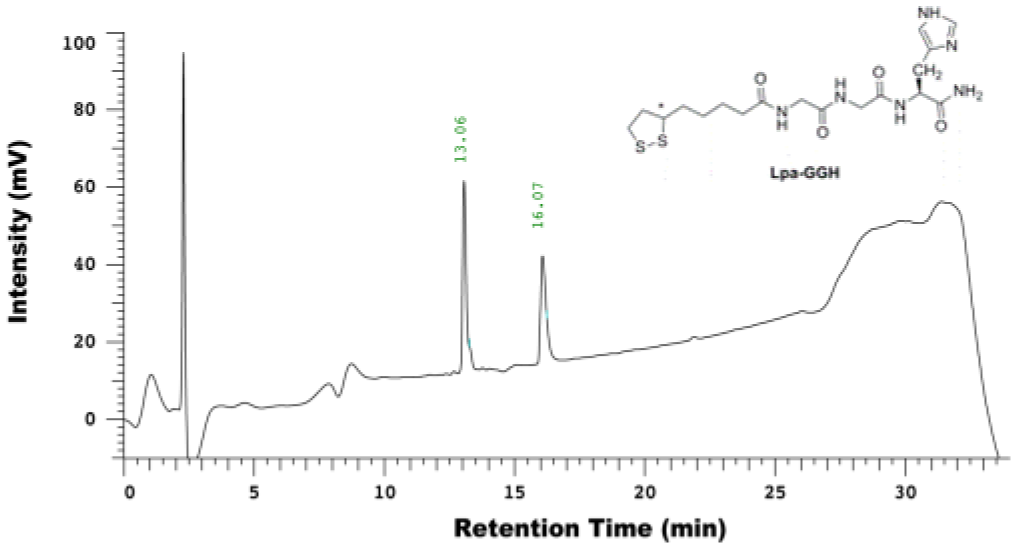

Figure S2: Analytical HPLC analysis of diasteomeric mixture of synthetic LPA-GGH

Reagents:

Fmoc-Rink- Amide-MB HA resin (Iris biotech GMPH), NMP(J.T.Baker), Fmoc-His(Trt)- OH (GL Biochem Ltd), Fmoc-Gly- OH (Genzyme), α-Lipoic acid (sigma), HATU(Luxembourg Bio technologies), DIPEA(Biolab), Piperidine(Biolab), TFA (Biolab), 1,2 Ethanedithiol (sigma), TIPS(Sigma)

X-Ray Phoelectron spectroscopy of Lpa-GGH SAM on Au substrates due to chelation with Cu^{+2}

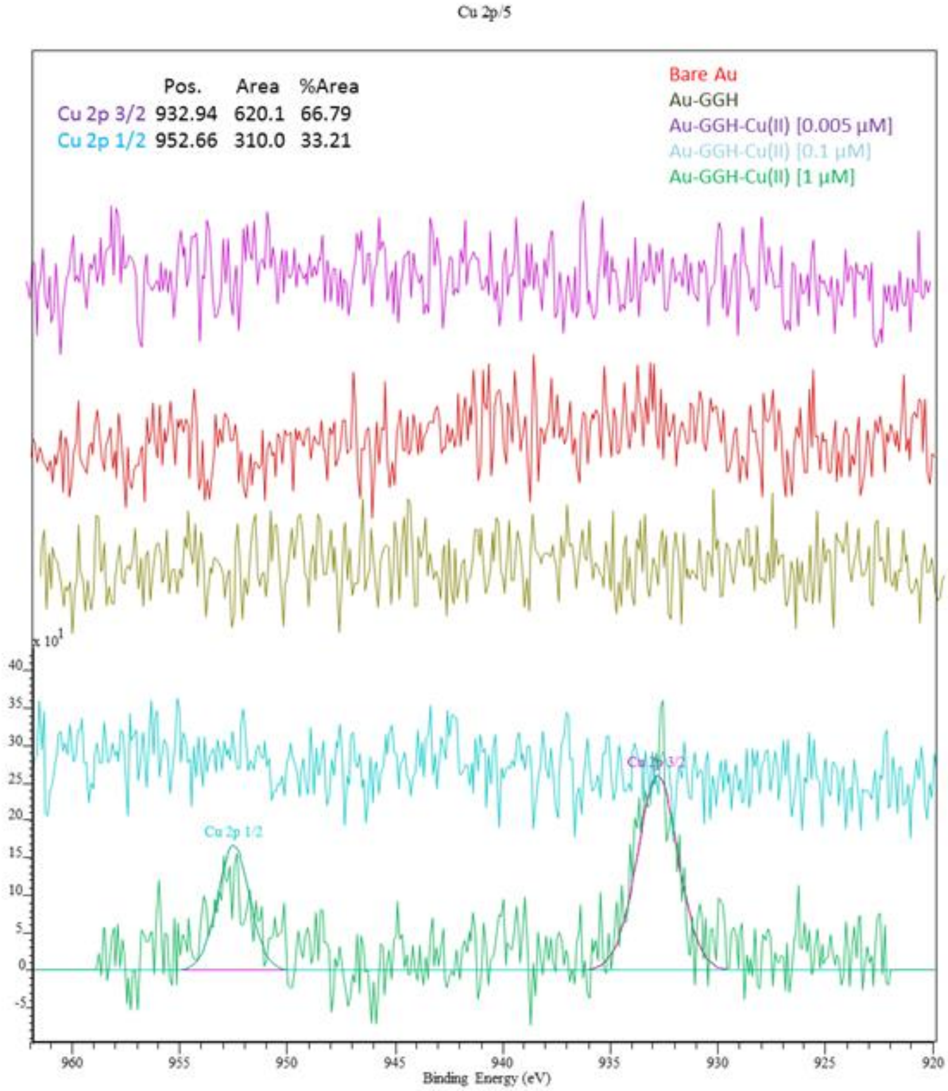

Figure S3. XPS analysis of Au-GGH SAM with Cu^{+2}. Cu 2p/5 integration

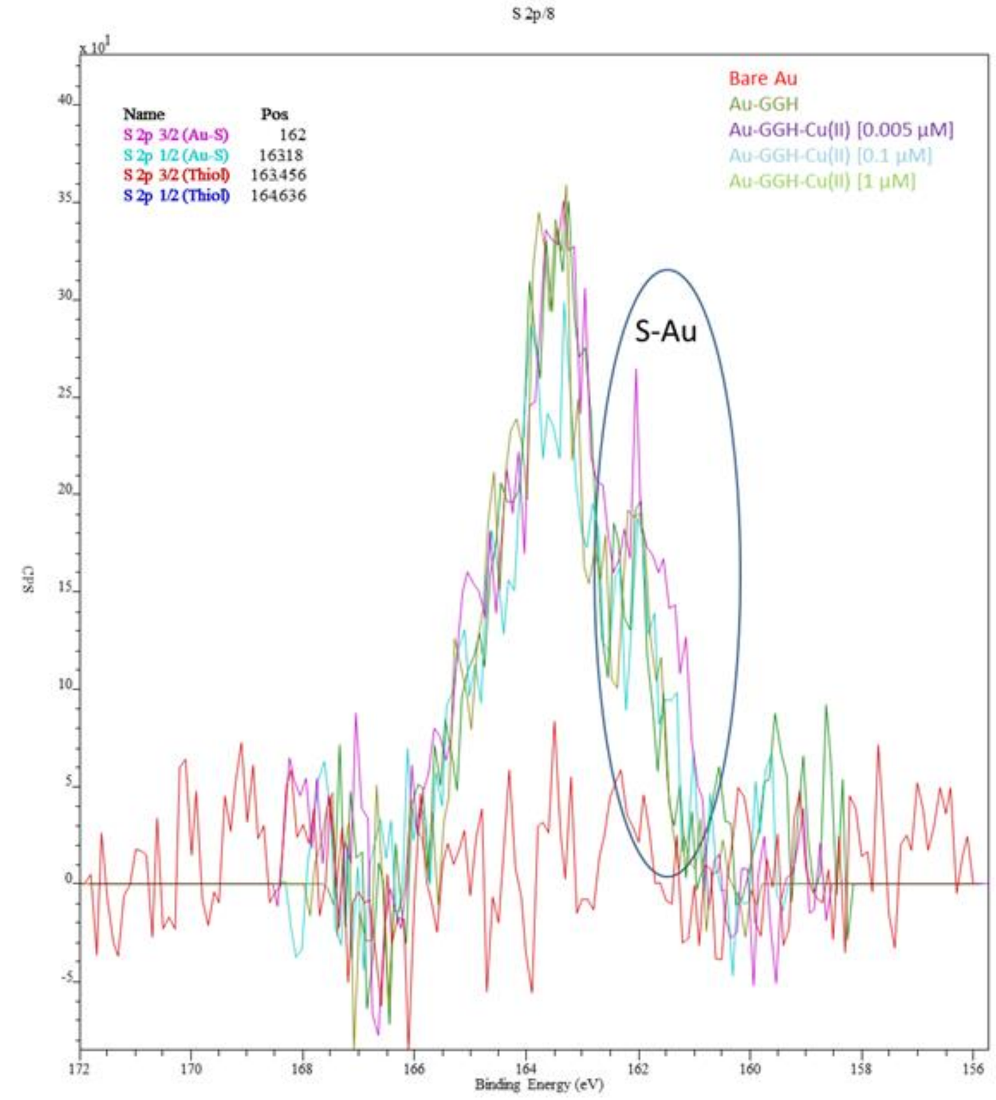

Figure S4. XPS analysis of Au-GGH SAM with Cu^{+2}. S 2p/8 integration
